# The 2020 Portrait of American Caregivers

**DOI:** 10.1093/geroni/igaa057.2372

**Published:** 2020-12-16

**Authors:** Regina Shih

**Affiliations:** RAND Corporation, Arlington, Virginia, United States

## Abstract

The prevalence of caregiving for an adult or child with special needs has increased significantly in the past five years (from 18.2% to over 21.3%), driven by an increase in the prevalence of caring for a family member or friend aged 50 and older. At the same time, care recipients have greater health and functional needs that necessitate care from others in comparison to 2015. These new 2020 data from the Caregiving in the US Survey by the National Alliance for Caregiving suggests that not only are more American adults taking on the role of caregiver, but they are doing so for increasingly complex care situations. This paper addresses the prevalence of caregiving including the demographics of family caregivers, relationship between the caregiver and the care recipient, health conditions of the care recipient, and living situations of care recipients and their caregivers.

